# Vegetation degradation impacts soil nutrients and enzyme activities in wet meadow on the Qinghai-Tibet Plateau

**DOI:** 10.1038/s41598-020-78182-9

**Published:** 2020-12-04

**Authors:** Jiangqi Wu, Haiyan Wang, Guang Li, Weiwei Ma, Jianghua Wu, Yu Gong, Guorong Xu

**Affiliations:** 1 College of Forestry, Gansu Agricultural University, Lanzhou, 730070 China; 2 School of Science and the Environment, Memorial University of Newfoundland, 20 University Drive, Corner Brook, NL A2H 5G4 Canada

**Keywords:** Ecology, Environmental sciences

## Abstract

Vegetation degradation, due to climate change and human activities, changes the biomass, vegetation species composition, and soil nutrient input sources and thus affects soil nutrient cycling and enzyme activities. However, few studies have focused on the responses of soil nutrients and enzymes to vegetation degradation in high-altitude wet meadows. In this study, we examined the effects of vegetation degradation on soil nutrients (soil organic carbon, SOC; total nitrogen, TN; total phosphorus, TP) and enzyme activities (i.e., urease, catalase, amylase) in an alpine meadow in the eastern margin of the Qinghai-Tibet Plateau. Four different levels of degradation were defined in terms of vegetation density and composition: primary wet meadow (CK), lightly degraded (LD), moderately degraded (MD), and heavily degraded (HD). Soil samples were collected at depth intervals of 0–10, 10–20, 20–40, 40–60, 60–80, and 80–100 cm to determine soil nutrient levels and enzyme activities. The results showed that SOC, TN, catalase and amylase significantly decreased with degradation level, while TP and urease increased with degradation level (*P* < 0.05). Soil nutrient and enzyme activity significantly decreased with soil depth (*P* < 0.05), and the soil nutrient and enzyme activity exhibited obvious "surface aggregation". The activities of soil urease and catalase were strongest in spring and weakest in winter. The content of TN in spring, summer, and autumn was significantly higher than observed in winter (*P* < 0.05). The soil TP content increased in winter. Soil amylase activity was significantly higher in summerm than in spring, autumn, and winter (*P* < 0.05). TP was the main limiting factor for plant growth in the Gahai wet meadow. Values of SOC and TN were positively and significantly correlated with amylase and catalase (*P* < 0.05), but negatively correlated with urease (*P* < 0.05). These results suggest the significant role that vegetation degradation and seasonal freeze–thaw cycle play in regulating enzyme activities and nutrient availability in wet meadow soil.

## Introduction

Soil organic carbon (SOC), total nitrogen (TN), and total phosphorus (TP) are the main nutrients in soil and are crucial to all biological processes^1^. On the one hand, vegetation growth and composition depend on the concentration of nutrients available in the soil^2^. On the other hand, the chemical composition of the plant residues and soil nutrient status affect microbial activity and microbial community structure, which in turn affect the decomposition rate of litter^3^. Therefore, the nutrient distribution characteristics of soil not only reflect the nutrient supply status and availability level in soil, but also have a profound influence on the composition of plant communities, and on the stability and health of ecosystem functions^4^. However, little is known about how the vegetation degradation affects the seasonal changes of soil nutrients and enzyme activities in the wet meadow of the Qinghai-Tibet Plateau.

Soil microorganisms play an important role in soil material transformation and are closely related to soil fertility and plant nutrition^5,6^. Soil enzyme are the catalyst of soil biological chemical reactions, and directly reflect soil metabolic demand and soil nutrient availability (one of the effective indexes to evaluate the soil fertility^7,8^ and plant growth). Additionally, plants can directly influence soil microbial composition and diversity by affecting rhizosphere secretions and soil oxygen content, and further indirectly affect enzyme activity^9^. Soil amylase and urease are the main biocatalysts for decomposition, turnover, and mineralization of soil organic matter, and are primary regulating factors of the soil carbon and nitrogen cycle^10,11^. Catalase can promote the decomposition of hydrogen peroxide into water and oxygen, and is an important redox enzyme system for soil humus synthesis and prevention of hydrogen peroxide toxicity to soil enzymes^12^. These soil enzyme activities are influenced by soil fertility level and the response of the soil to environmental changes. Therefore, enzyme activity is used as a sensor to evaluate soil fertility status^13^ due to the integration of information on microbial status and soil physical and chemical conditions^14,15^.

Vegetation is a key component of terrestrial ecosystems^16^. Vegetation degradation is regarded as a decrease in biomass or a change in the structure of the vegetation community. Vegetation degradation can be caused by climate change and human activities, such as climate warming, precipitation change, and overgrazing^17–19^. Vegetation degradation plays an essential role in changes in soil nutrient and enzyme activities. Previous studies have shown that vegetation degradation significantly reduced biomass and soil carbon content^20,21^. Moreover, vegetation degradation increased soil nitrite content and the number of ammonia-oxidizing microorganisms, thereby further exacerbating the risk of soil nitrogen loss^22^. This reduction in nutrient inputs decreases the microbial diversity in the soil and the resistance of the soil to environmental stress^23^, and has the potential to change the enzyme activities. However, the response of soil enzyme activity to vegetation degradation in wet meadows and the relationship between soil nutrients and enzyme activity are not well understood.

The Qinghai-Tibet Plateau (QTP) wetland has an average elevation of 4000 m. It is one of the concentrated areas of plateau wetland distribution across China. Climate change here has been more significant and advanced compared with other areas^24^. In recent years, the mean annual temperature in the QTP has increased at a rate of 0.16 °C per decade^25^, and at the same time grazing intensity has exceeded the theoretical grazing capacity of the ecosystem^26^, resulting in many wetlands that have degraded or disappeared^27^. For example, the wet meadows have been transformed into grassland meadows and ultimately sandy meadows at severely deteriorated sites^25^. At the same time, vegetation degradation has caused a significant decrease in plant coverage and biomass in wet meadows, aggravated soil erosion and soil nutrient loss, led to soil degradation, and further affected ecological functions^28^. Studies have shown that vegetation degradation reduces the nutrient input in plant biomass, resulting in a decline in SOC and TN levels in peatlands^29^. The research on the vegetation degradation of QTP wet meadow mainly focuses on greenhouse gas emissions, microbial activity in the root area, and SOC^30–32^. Furthermore, several time periods are of special importance and concern for wet meadows in this region, including the spring–autumn-winter period when plant growth is slow or non-existent, and lower soil temperatures affect microbial activity^33^. During these periods, the leaching potential of N and P is the greatest, and frequent temperature changes will affect soil nutrient processes^34,35^. However, few have examined how the soil nutrients and enzyme activities changes in the wet meadows due to vegetation degradation, especially in QTP. Therefore, in order to better predict soil biogeochemical processes in QTP, the changes in soil nutrients and enzyme activities under different intensities of vegetation degradation in the QTP wet meadow require further investigation.

Thus, the primary aim of this study was to quantify the distribution of soil nutrients and enzyme activities to a soil depth of 1 m in a QTP wet meadow and how they are affected by vegetation degradation. Four vegetation degradation level were selected: primary wet meadows (CK), lightly degraded (LD), moderately degraded (MD), and heavily degraded (HD). We measured the contents of soil nutrient (i.e., SOC, TN, TP) and enzyme activities (i.e., catalase, amylase and urease) during the spring–summer–autumn–winter (2018–2019). Three specific objectives were established: (1) characterize the differences in the soil nutrients and enzyme activities among the various levels of vegetation degradation; (2) quantify the nutrients and enzyme activities in different soil layers (0–10, 10–20, 20–40, 40–60, 60–80, and 80–100 cm); and (3) analyze the correlation among soil nutrients and enzyme activities. We hypothesized that with increasing vegetation degradation degree, (1) the contents of soil nutrients (i.e., SOC, TN, and TP) will decrease because of the lower litter input and higher nutrients decomposition after vegetation degradation; (2) the decline in soil nutrients will decrease the enzyme activities (i.e., catalase, amylase and urease); and (3) with the increase of soil depth, the effects of vegetation degradation on soil nutrients and enzyme activities will be weakened gradually because the plant underground biomass (root system) gradually decreases over depth.

## Materials and methods

### Study area

The Gahai wet meadow is located in Gahai-Zecha National Nature Reserve, Luqu County, Gansu Province, at the northeast margin of Qinghai-Tibet Plateau (33°58′12″–34°32′16″ N, 102°05′00″–102°47′39″ E). According to the second National Wet Meadow Survey, the wet meadow area in the reserve is 5.78 × 10^4^ ha, with the marsh meadow accounting for 88.4% (5.12 × 10^4^ ha) of the total wet meadow area. This region belongs to the Qinghai-Tibet Plateau climatic zone (alpine cold humid climate area) with average annual temperature of 2.9 °C. The July maximum average temperature of 12.9 °C is 21.4 °C higher than the January minimum average temperature of − 8.5 °C^28^. In 2018, the precipitation was 770 mm (mainly concentrated mainly from May to September) and the annual evaporation was 1150 mm. Soil types include mainly dark meadow soil, marsh soil, and peat soil.

### Experimental design

**Figure 1:**
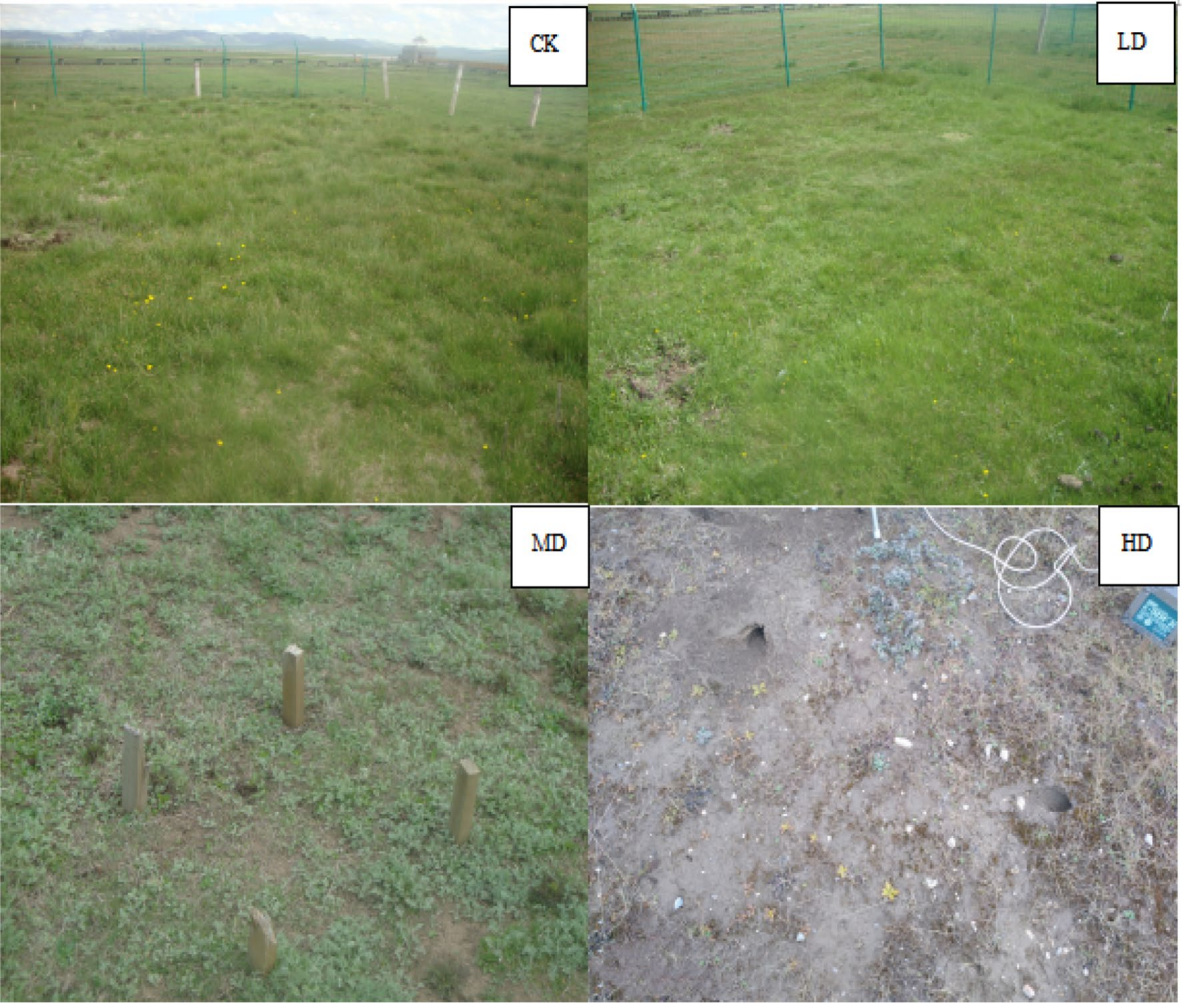
General characteristics of sampling sites of the Gahai wet meadow, China. CK = primary wet meadow; LD = lightly degraded; MD = moderately degraded; HD = highly degraded.

Surface vegetation scarcity is the obvious characteristic of wet meadow degradation. Vegetative cover, dominant species, and biomass were identified as key indicators for assessment of wetland degradation levels^33^. Vegetation characteristics of the Gahai wet meadow are influenced by groundwater level, distance from the lake, composition of dominant species, vegetation cover, and aboveground biomass. In the 1950s, grazing intensity exceeded the theoretical grazing capacity of the ecosystem and led to vegetation degradation^25^, with four vegetation degradation levels commonly and widely recognized in other studies for wet meadow on the QTP^30,32^. These four degradation levels are primary wet meadow (CK), lightly degraded (LD), moderately degraded (MD), and heavily degraded (HD) (Table 1 and Fig. 1). Three replicated plots of 10 m × 10 m for each degradation level were established in early May 2013 and maintained for five years until soil sampling. To reduce potential edge effects, we maintained a buffer zone of at least 5 m between any plots. We collected soil samples in four different seasons: spring, summer, autumn, and winter^36^. These occurred on 20 April (spring), 18 July (summer) and 25 October (autumn) of 2018, and on 25 January (winter) of 2019.

**Table 1:** General characteristics of sampling sites of the Gahai wet meadow, China.

Degradation level^a^	Water table (cm)	Distance from Lake (m)	Dominant species composition	Coverage (%)	Biomass (dry matter) (g m^−2^)
CK	10.5 ± 0.95	25	*Carex meyeriana* + *Potentilla anserina* L + *Poa subfastigiata Trin*	96	2133.34
LD	− 0.6 ± 1.41	50	*Carex moorcroftii* + *Artemisia frigida* Willd + *Oxy tropis sp*	86	1378.23
MD	− 40.7 ± 2.25	80	*Artemisia sacrorumvar. Messerschmidtiana* *Y.R.Ling* + *Artenisia subulata Nakai*	45	347.86
HD	− 100.6 ± 4.08	120	Low groundwater level, overgrazing, and severe rodent damage led to near zero vegetation coverage		

^a^CK, primary wet meadow; LD, lightly degraded; MD, moderately degraded; HD, highly degraded.

### Soil sampling

Soil samples were collected in each of the twelve plots after removing dead leaves from the surface layer (20 April, 18 July, 25 October 2018 and 25 January 2019). Samples were obtained by using an auger (diameter 50 mm) at seven points in each plot (two points near opposite sides of the plot and three points along a diagonal across the plot (“Z” pattern). Each of the seven samples collected in each plot was divided into six sampling intervals (0–10, 10–20, 20–40, 40–60, 60–80, and 80–100 cm) resulting in 4 × 3 × 6 × 7 = 504 samples. Samples (4 × 3 × 6 = 72) from the same soil depth interval were combined to form a mixed soil sample that was taken back to the laboratory for analysis.

### SOC and nutrient analysis

The SOC content was determined by the Walkley–Black potassium dichromate oxidation method^32^. The soil sample (0.1 g, accurate to 0.001 g) was extracted with 7.5 ml of 0.4 M K_2_Cr_2_O_7_ and 7.5 ml of concentrated H_2_SO_4_ at 180 °C for 30 min. The soil sample (1 g, accurate to 0.001 g) was digested with 5 ml of concentrated H_2_SO_4_ at 400 °C. When the color of the extracting solution became milky white, heating was stopped and the whole solution was transferred to a 100 ml volumetric flask. 5 ml of the solution was used to measure total nitrogen with the Semi-Micro Kjeldahl method^37^. 10 ml of the solution was used to determine total phosphorus with the Mo-Sb colorimetric method^38^. All analyses were done within one week of soil sampling.

### Enzyme activity analysis

The urease activities were analyzed using the methods presented by Guan, Yin and Ge^39–41^. Soil (2 g) was treated with 10 ml urea (10%), 20 ml citrate buffer (1 M, pH 6.7), and 1 ml methylbenzene and kept at room temperature for 15 min. The sample was then shaken at 37 °C for 24 h. The solution was filtered, and 1 ml of the filtrate was mixed with 20 ml distilled water, 4 ml sodium phenolate hydroxide, and 3.0 ml sodium hypochlorite. The NH_4_^+^-N was determined 20 min later by Spectrophotometer at 578 nm. Urease activity was expressed in milligram of NH_4_^+^-N per gram of soil released in 24 h.

The amylase enzyme activities were analyzed using the methods presented by Guan and Xie^39,42^. With the method of 3, 5-dinitrosalicylic acid, the amylase activities were measured using soluble starch as substrate, respectively. These measurements were expressed as mg glucose (g soil 24 h)^−1^ for amylase activities.

Soil catalase activity was determined by potassium permanganate titration^43,44^. 40 ml of distilled water and 5 ml (3%) hydrogen peroxide solution were added to 2 g soil, which was shaken for 30 min and then filtered. We then took 25 ml of the filtrate and titrated it to pink with 0.1 M potassium permanganate. For all enzymes, three replicates of each subsample were analyzed, and substrate-free and soil-free controls were added for each sample to account for non-enzymatic substrate hydrolysis.

### Statistical analysis

One-way analysis of variance (ANOVA) using SPSS 19.0 software (https://www.ibm.com/products/spss-statistics) was used to determine relationships between vegetation degradation, soil depth, seasonal variation and SOC, TN, TP, and enzyme activity. Pearson correlation analysis and linear regression were used to examine the relationships between soil catalase, invertase, urease, and soil nutrient (i.e., SOC, TN, TP). Differences in treatment means were analyzed using Duncan’s multiple range test at a significance level of 95% (*P* < 0.05). A repeated-measures ANOVA was used to test the effects of vegetation degradation on nutrient (SOC, TN, and TP) and enzyme activity (urease, catalase, and amylase) using season as the repeated variable.

## Results

### Vertical and seasonal variation of SOC content under vegetation degradation

**Figure 2:**
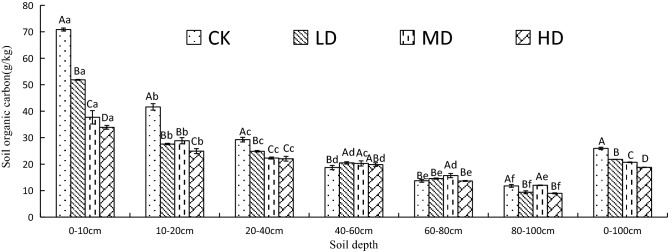
Vertical variation of soil organic carbon under four vegetation degradation levels in the Gahai wet meadow, China. CK = primary wet meadow; LD = lightly degraded; MD = moderately degraded; HD = highly degraded. Error bars indicate standard errors of the mean (n = 3). Different capital letters (A, B, C, D) indicate significant differences in SOC due to vegetation degeneration (*P* < 0.05); different lower case letters (a, b, c, d, e, f) indicate significant differences between the soil layers under the same vegetation degradation (*P* < 0.05).

The average SOC content in the 0–100 cm layer of CK degradation level was 18.99%, 25.25%, and 38.01% higher than that of LD, MD, and HD, respectively. The SOC content decreased with increasing soil depth under the four degradation levels (Fig. 2). The SOC in the 0–40 cm layer accounted for 65.9% (CK), 59.3% (LD), 53.7% (MD), and 54.8% (HD) of the SOC in the 0–100 cm soil profile. The SOC showed significant differences in the 0–40 cm layer among the four degradation levels (*P* < 0.05).

**Figure 3:**
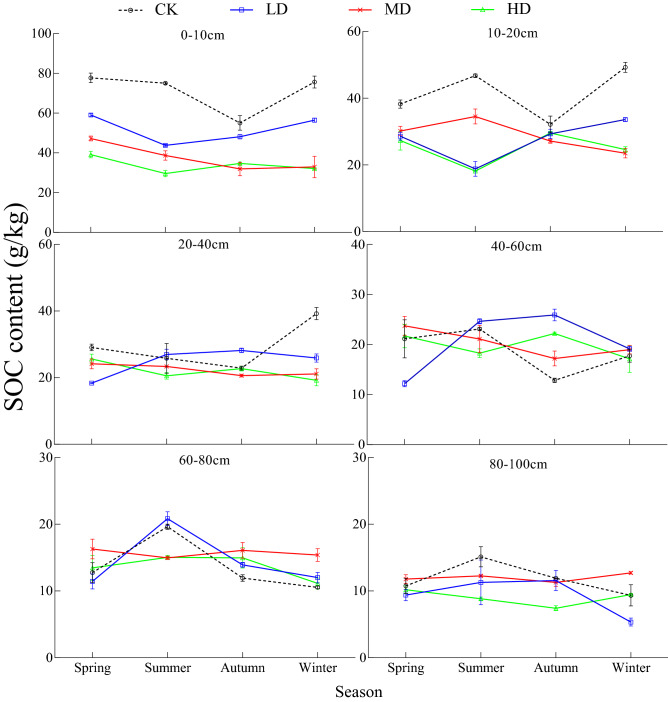
Seasonal variation of SOC content under different vegetation degradation levels in the Gahai wet meadow, China. CK = primary wet meadow; LD = lightly degraded; MD = moderately degraded; HD = highly degraded. Error bars indicate standard errors of the mean (n = 3).

SOC in the four degraded meadow wetlands showed obvious seasonal variation (Fig. 3), with more pronounced seasonal variation in the topsoil layers (0–10 and 10–20 cm) than in the subsoil layers (20–40, 40–60, 60–80, and 80–100 cm). In the 0–10 cm layer, the SOC of CK and LD showed a "decreasing then increasing" trend with the seasonal changes, while the SOC of MD and HD showed a gradually decreasing trend. In the 10–20 cm layer, the maximum SOC contents of CK and LD occurred in winter, while the maximum SOC contents of MD and HD appeared in summer and autumn. The repeated-measures ANOVA showed significant interactions between season and vegetation degradation on SOC contents in all sample layers except for the 40–60 cm layer (Table S1).

### Changes of soil TN and TP content under vegetation degradation

**Figure 4:**
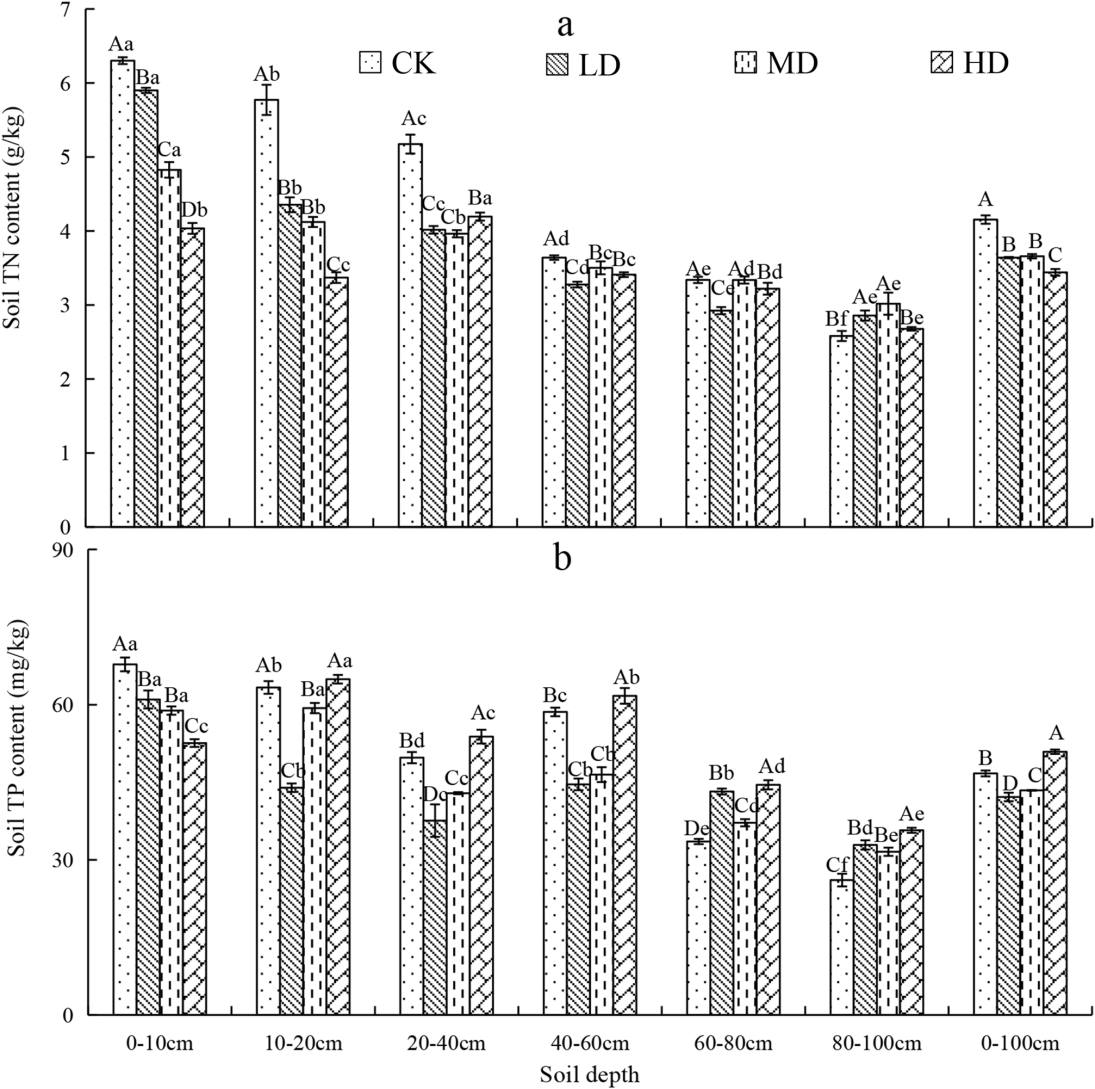
Vertical variation of soil TN (**a**) and TP (**b**) content under different vegetation degradation levels in the Gahai wet meadow, China. CK = primary wet meadow; LD = lightly degraded; MD = moderately degraded; HD = highly degraded. Error bars indicate standard errors of the mean (n = 3). Different capital letters (A, B, C) indicate significant differences in SOC due to vegetation degeneration (*P* < 0.05); different lower case letters (a, b, c, d, e, f) indicate significant differences between the soil layers under the same vegetation degradation (*P* < 0.05).

The TN content of CK soil in the 0–100 cm soil profile was 14.0%, 13.4%, and 20.6% higher than that of MD, LD, and HD, respectively (*P* < 0.05, Fig. 4a). The TP content in the 0–100 cm soil profile of the Gahai wet meadow followed the order of HD > CK > MD > LD (*P* < 0.05, Fig. 4b). TN and TP decreased with increasing soil depth under all four degradation levels.

**Figure 5:**
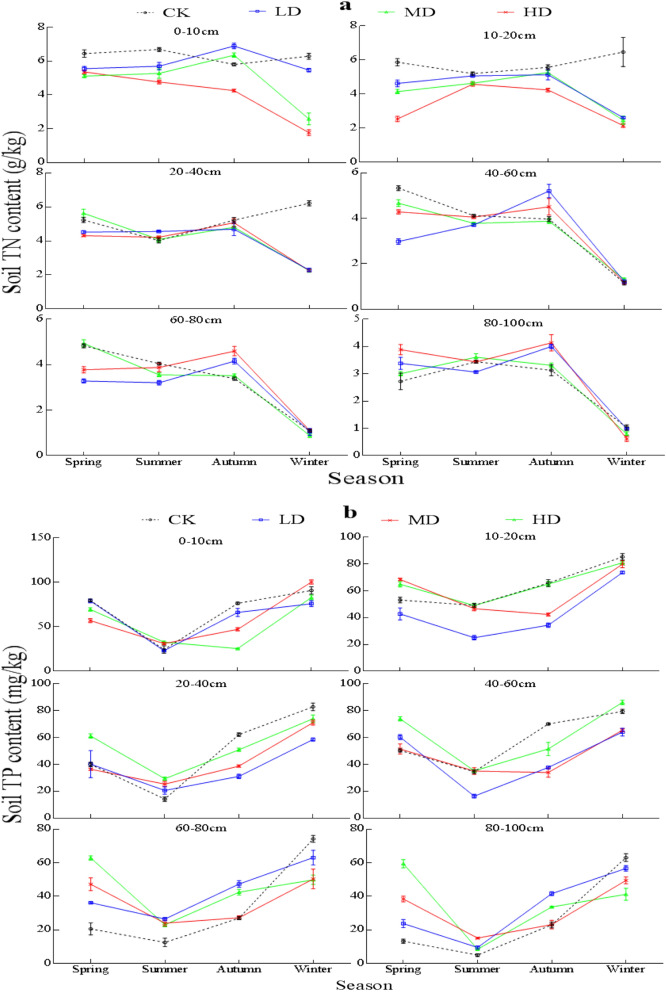
Seasonal variation of soil TN (**a**) and TP (**b**) under different vegetation degradation levels in the Gahai wet meadow, China. CK = primary wet meadow; LD = lightly degraded; MD = moderately degraded; HD = highly degraded. Error bars indicate standard errors of the mean (n = 3).

TN and TP for the four degradation levels showed significant seasonal variation (Fig. 5). There were significant interactions between season and vegetation degradation in TN and TP for all sample layers (Table S1). With the change of season, the soil TP content was the lowest in summer and gradually increased in autumn and winter (Fig. 5b). In contrast, TN content showed a very different variation pattern form than observed for TP over time (Fig. 5a).

### Vertical variation of soil enzyme activity under vegetation degradation

**Figure 6:**
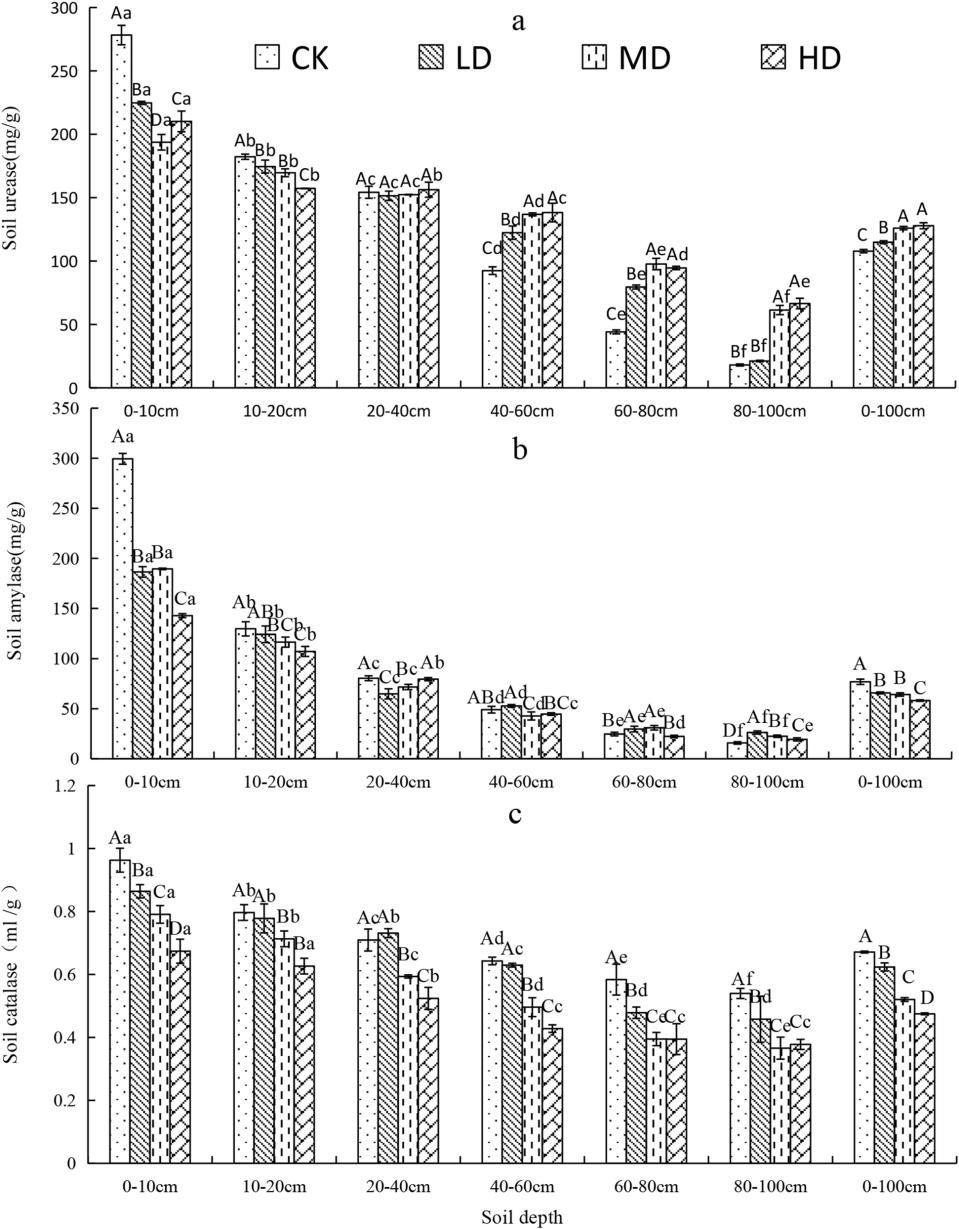
Vertical variation of soil urease (**a**), amylase (**b**), and catalase (**c**) for different levels of vegetation degradation in the Gahai wet meadow, China. CK = primary wet meadow; LD = lightly degraded; MD = moderately degraded; HD = highly degraded. Error bars indicate standard errors of the mean (n = 3). Different capital letters (A, B, C, D) indicate significant differences in soil urease, amylase, and catalase due to vegetation degeneration (*P* < 0.05); different lower case letters (a, b, c, d, e, f) indicate significant differences between the soil layers under the same vegetation degradation (*P* < 0.05).

Soil urease activity in the 0–100 cm soil profile was significantly higher for MD and HD than for CK and LD (Fig. 6a). Soil amylase activity in the 0–100 cm soil profile of CK was 16.9%, 19.8%, and 32.2% higher than that of LD, MD, and HD (*P* < 0.05, Fig. 6b). Soil catalase activity in the 0–100 cm soil profile of the Gahai wet meadow followed the order of CK > LD > MD > HD (Fig. 6c). With increasing soil depth, soil enzyme (urease, amylase and catalase) activities under the four vegetation degradation levels decreased. Urease activity in 0–20 cm layer of CK, LD, MD, and HD accounted for 42.7%, 34.8%, 28.8%, and 28.7%, respectively, of urease activity in entire 0–100 cm soil profile. Soil amylase activity in the 0–20 cm soil layer accounted for 55.8.%, 47.2%, 47.7%, and 43.0% of amylase activity in the 0–100 cm soil layer for CK, LD, MD, and HD vegetation degradation levels, respectively.

### Seasonal variation of soil enzyme activity under vegetation degradation

**Figure 7:**
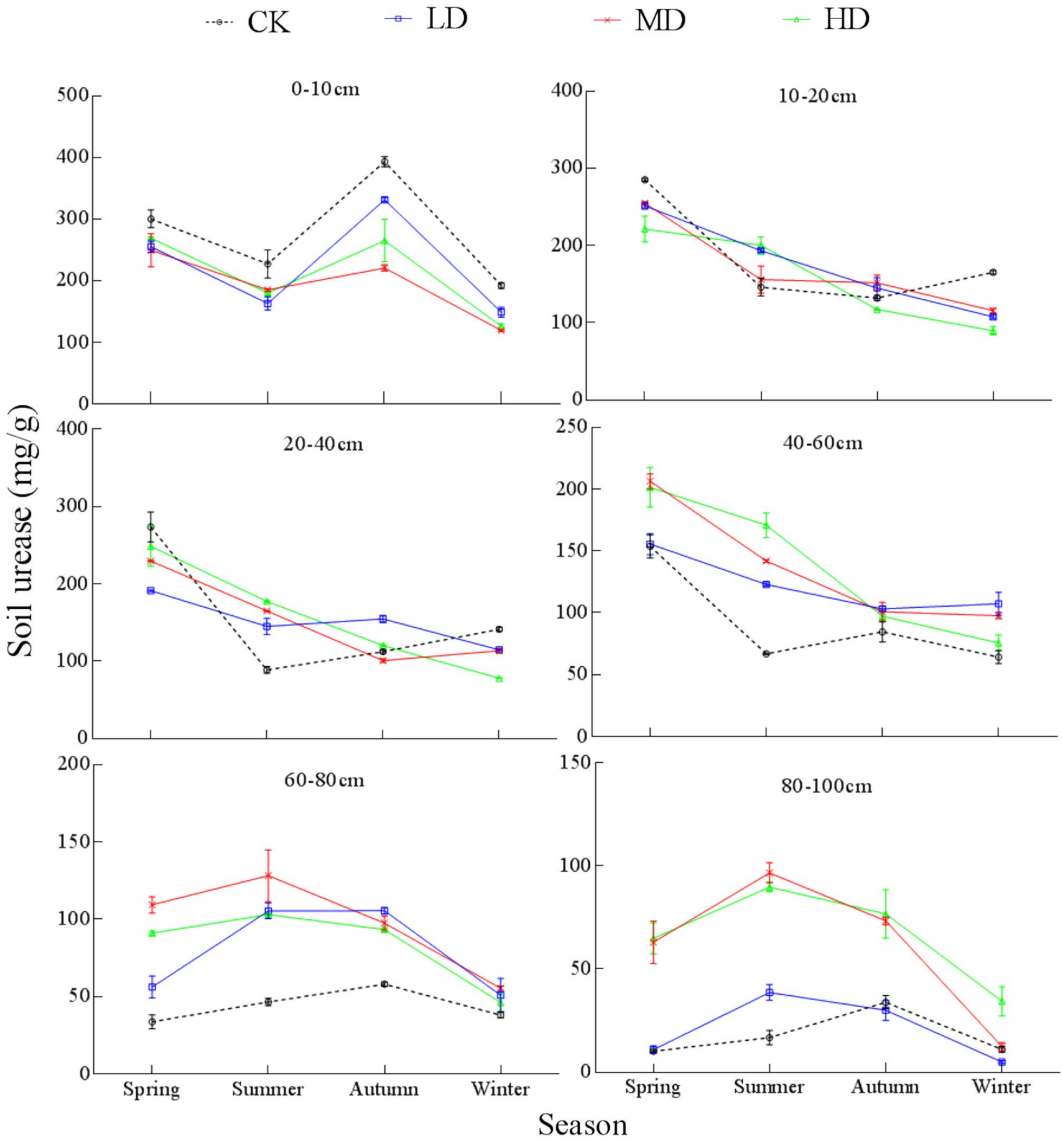
Seasonal changes of soil urease under different levels of vegetation degradation in the Gahai wet meadow, China. CK = primary wet meadow; LD = lightly degraded; MD = moderately degraded; HD = highly degraded. Error bars indicate standard errors of the mean (n = 3).

Urease activity showed obvious seasonal variation under the four vegetation degradation levels (Fig. 7). The repeated-measures ANOVA showed (except for the 20–40 cm layer) significant interactions between season and vegetation degradation in the urease activity in all sample layers (Table S2). Urease activity for CK and LD in the 0–100 cm soil profile showed a "down-up-down" change pattern with time, while urease activity of MD and HD showed a gradual downward trend with time. Urease activity of the 0–10 cm layer showed a "double peak" change pattern with time, while urease activity of the 10–60 cm layer decreased with time, and urease activity of the 60–100 cm layer showed an "up-down" change pattern.

**Figure 8:**
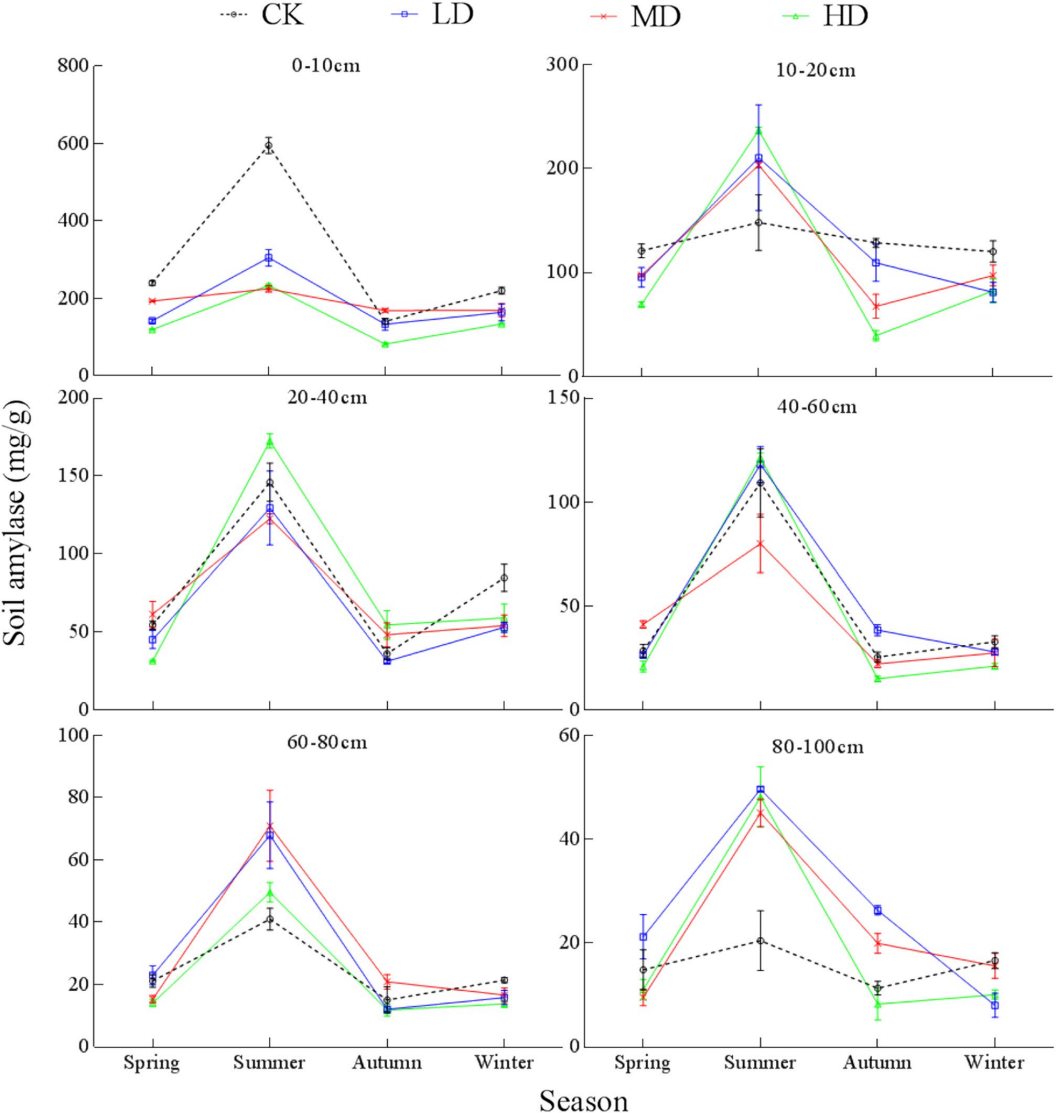
Seasonal changes of soil amylase under different levels of vegetation degradation in the Gahai wet meadow, China. CK = primary wet meadow; LD = lightly degraded; MD = moderately degraded; HD = highly degraded. Error bars indicate standard errors of the mean (n = 3).

Amylase activity in the 10–20 cm soil layer of CK followed a "single peak" change pattern while the change pattern in other soil layers could be characterized as having a "double peak". Maximum amylase activity for LD appeared in summer, and the maximum values in the 0–10, 10–20, 20–40, 40–60, 60–80, and 80–100 cm layers were 2.29, 2.60, 4.13, 4.45, 5.62, and 6.17 times the minimum values, respectively. Amylase activity in the 0–60 cm layer for MD increased to the maximum in summer, and the minimum appeared in autumn. Amylase activity in the 60–100 cm layer was "unimodal", and the minimum occurred in spring (Fig. 8). There were significant interactions between season and vegetation degradation in amylase activity in all sample layers (Table S2).

**Figure 9:**
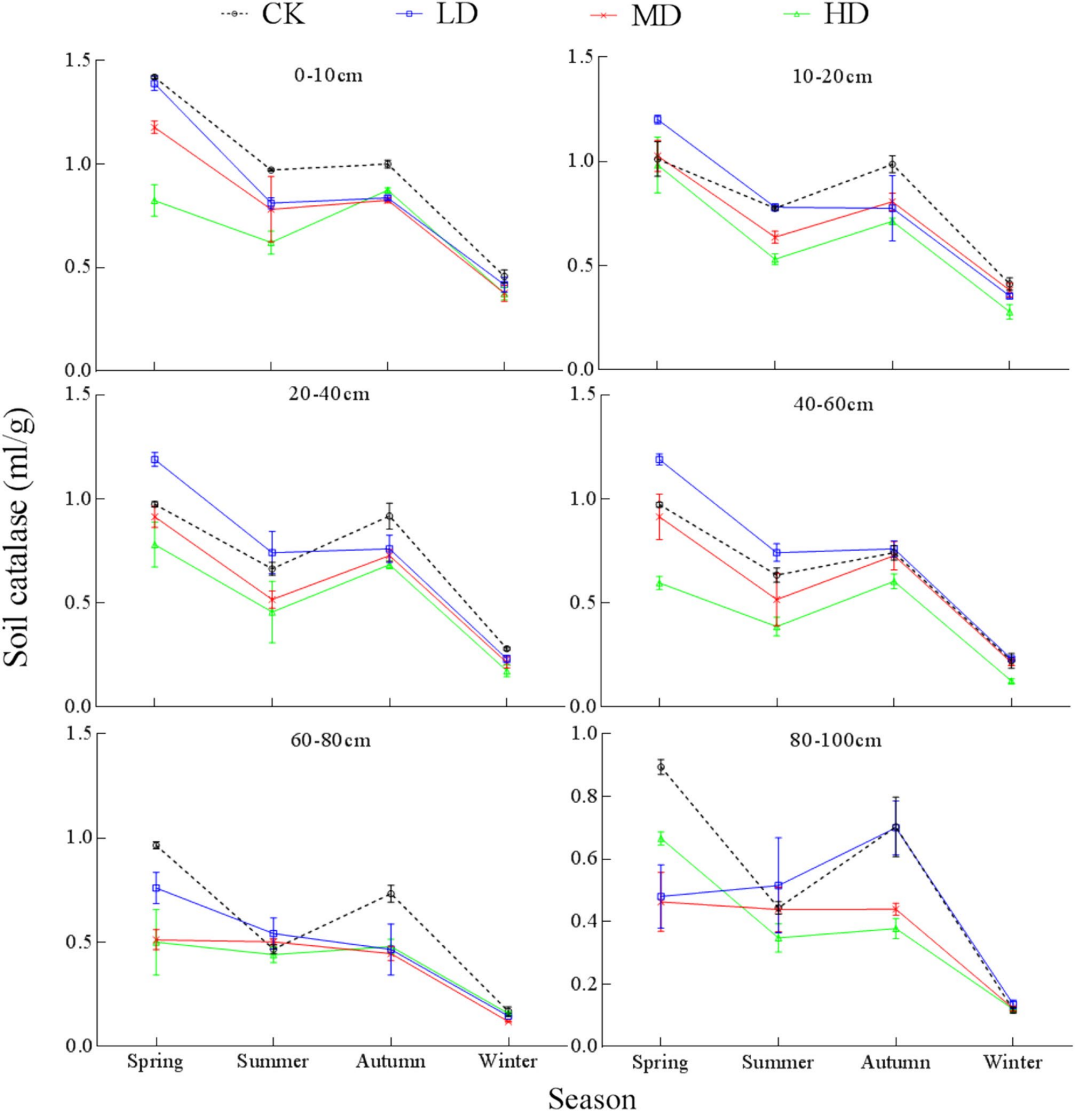
Seasonal changes of soil catalase under different levels of vegetation degradation in the Gahai wet meadow, China. CK = primary wet meadow; LD = lightly degraded; MD = moderately degraded; HD = highly degraded. Error bars indicate standard errors of the mean (n = 3).

Soil catalase activity in the 0–100 cm soil profile showed a "double peak" change pattern over time (Fig. 9) for all four degradation levels, with the highest activity in spring, a slight decrease in summer, followed by a rise in autumn, and with the lowest activity observed in winter. Catalase activity of CK in the 0–100 cm soil profile in spring was 62.2%, 22.36%, and 308.8% higher than the activity in summer, autumn, and winter, respectively. For LD, catalase activities in the 0–100 cm soil profile in spring were 49.8%, 43.1%, and 342.3% higher than that in summer, autumn and winter, respectively. For MD, catalase activities in the 0–100 cm soil profile in spring were 86.3%, 58.3%, and 376.1% higher than in summer, autumn and winter, respectively. For HD, catalase activities in the 0–100 cm soil profile in spring were 126.5%, 70.1%, and 453.1% higher than in summer, autumn and winter, respectively. There were also significant interactions between season and vegetation degradation in catalase activity in all sample layers (Table S2).

### Correlation between soil nutrients and enzyme activities

Correlations among soil nutrients and enzyme activities across the four vegetation degradation levels were analyzed at a depth of 0–100 cm (Table 2). Significantly positive correlations were observed among SOC and catalase, amylase activity (r = 0.951, 0.966, respectively, *P* < 0.05), and significantly negative correlation was found between SOC and urease (r = − 0.928, *P* < 0.05). TN was negatively correlated with urease (r = − 0.835, *P* < 0.05), and significantly positive correlations were detected among TN and catalase and amylase activity (r = 0.823 and 0.960, respectively, *P* < 0.05). In addition, TP was negatively correlated with all enzyme activities except urease (*P* > 0.05).

**Table 2:** Correlation between soil nutrients and enzyme activities at a depth of 0–100 cm across the four vegetation degradation levels.

	SOC	TN	TP	Urease	Catalase	Amylase
SOC	1					
TN	0.975*	1				
TP	− 0.248	− 0.158	1			
Urease	− 0.928*	− 0.835*	0.300	1		
Catalase	0.915*	0.823*	− 0.430	− 0.975*	1	
Amylase	0.966*	0.960*	− 0.280	− 0.878*	0.880*	1

SOC, soil organic carbon; TN, total nitrogen; TP, total phosphorus.

* indicates significant correlation at *P* < 0.05.

## Discussion

### Effects of vegetation degradation on soil nutrients

SOC, TN, and TP are very important ecological factors which can significantly affect the productivity of wetland ecosystems^45^. This study showed that vegetation degradation has a significant impact on wetland SOC (Fig. 2). The SOC comes mainly from the humification process of litter residues and the rhizosphere secretions released from the rhizosphere during plant growth^46,47^. Aboveground biomass decreases as vegetation degradation intensifies (Table 1), resulting in a decrease in litter and the amount of plant residues that can enter the soil, resulting in a decline in the main source of wetland SOC^48,49^. This result confirmed part of our first hypothesis about SOC dynamics. The SOC content decreases with soil depth, and it is mainly concentrated in the 0–20 cm layer, which confirmed part of our third hypothesis. Some studies have shown that the vegetation influence on the SOC content was the greatest in the topsoil (0–20 cm)^50^. Generally, high SOC content in the topsoil can be attributed to vegetation litter inputs at the surface, and vegetation root decomposition, especially because the maximum wet meadows rooting depth is 20 cm^51^. Despite having a higher SOC content in the surface, litter availability and the decomposition rate have been reported to decrease with increasing soil depth^52,53^, which leads to a lower SOC content with increasing soil depth.

Our research showed that the SOC content has complex seasonal changes (Fig. 3), which was consistent with previous studies^54,55^. The soil SOC content was higher in spring and winter, and lower in summer and autumn, and mainly concentrated in the surface layer (0–10 and 10–20 cm). These results may be attributed to the following reasons. On the one hand, higher soil temperature and water content in summer and autumn may promote the decomposition of soil carbon, resulting in more SOC emitted as CO_2_ or CH_4_ into the atmosphere^56^. On the other hand, surface litter in spring and winter increases the amount of carbon resources returned to the soil^57,58^, resulting in accumulation of SOC, especially in topsoil layers because the maximum rooting depth in wet meadows is 0–20 cm^51^. Moreover, the complex seasonal changes in SOC content may also be affected by soil properties and enzyme activity^59^. It may be the reason why we found a significant positive correlation between SOC content and TN and enzyme activity (Table 2).

Soil TN content in the 0–100 cm gradually decreased with vegetation degradation level (Fig. 4a), which verified part of our first and third hypothesis about soil TN dynamics. This may be due to the decrease of aboveground biomass, litter, and soil nutrient content with increasing degradation level, as well as decreases in oxygen content and microbial biomass that occur with increasing soil depth. On the one hand, these decreases are not conducive to the development of soil structure, and result in a slowing of nutrient cycling^60,61^. On the other hand, urease participates in the cycle of organic nitrogen in the soil and makes nitrogen available for plants^62^. Plants absorb soil nitrogen during growth, leading to decreased soil TN content. This may be the reason why we detected the negative correlation between soil TN and urease activity (Table 2). In addition, the litter on the surface of CK plots increases the source of soil nitrogen under decomposition by microorganisms^63^. Coupled with the remediation of plants and root exudates to the soil and environment^64,65^, the CK soil nitrogen accumulation rate is greater than the plant absorption rate, resulting in significantly higher TN contents in the surface soil of CK plots than in the other vegetation degradation plots.

Soil TP in the 0–100 cm soil profile with HD was significantly higher than seen for the other three degradation levels (Fig. 4b), which falsified part of our first hypothesis about soil TP dynamics, but still confirmed our third hypothesis about vertical change of soil TP dynamics. In the process of plant growth, phosphorus in soil is absorbed for root growth^2^. With the decrease of vegetation biomass (Table 1), the content of phosphorus absorbed decreases^66^, thereby increasing the content of TP in soil. Additionally, rodent damage is a serious problem for heavily degraded wet meadows^67^. In the process of burrowing, rodents destroy the original structure of the soil^68^, thereby increasing soil temperature, and reducing the water content of the soil layer. This may cause serious water stress for microbial activities and thus reduce microbial activity. As a result, the mineralization and decomposition rate of soil phosphorus decreased with the decrease of soil microbial activity^69^, and ultimately could result in an accumulation of TP in the soil of the HD plot. However, the mechanism of building up a higher TP in the soil of the CK plot is different. The higher TP content of the CK plot originated primarily from litter and root exudates^70^, where the return of residue P to the soil is much greater than the P absorbed by plants to support their growth. Studies have shown that when soil TP becomes the limiting factor, plant root exudates ionize H^+^ through organic acids or activate insoluble inorganic P in the soil through exchange and reduction to increase the biological effectiveness of P^71^. Because the higher organic carbon content of CK soil provides sufficient energy for microorganisms, the higher microbial activity increases the decomposition rate of surface litter. The result is that the phosphorus in the decomposition products of litter is returned to the surface soil by leaching^72^, and total phosphorus content of CK soil increases further.

There were obvious seasonal changes of soil nutrients in the four degradation levels. TP content reached minimum values in summer, rose in autumn, and reached maximum values in winter (Fig. 5b). Plants and soil organisms normally have a period of vigorous growth during the summer under conditions of increased rainfall and soil temperature. As vegetation extracts P from the soil for the synthesis of its own substances^59^, and additional P is lost due to leaching by rainwater^73^, TP content of the soil drops to the lowest values in the summer. The perennial plants in our plots stopped growing or even withered after autumn, thereby reduceing the rate of phosphorus absorption in the soil. Litter decomposition increased the source of soil nutrients^74^, so that TP content in autumn and winter increased. Changes in TN and TP under the four degradation levels were opposite (Fig. 5a). The temperature gradually increased in spring, and increases the soil microbial activity, litter decomposition rate, and soil TN content increased concurrently. In addition, previous studies have shown that soil nitrogen mineralization is still active in winter^75^, and the activity of microorganisms such as ammonia-oxidizing bacteria and ammonia-oxidizing archaea in soil is still high^76^, resulting in consumption a significant amount of TN, and thus a decrease the TN in winter. TP is one of the main factors affecting soil nitrogen mineralization. A higher TP content in the soil can promote the process of nitrogen mineralization^77,78^, that increases the amount of soil nitrogen leaching loss in winter and leads to TN content being the lowest in winter.

### Effect of vegetation degradation on soil enzyme activity

Soil enzyme level are an indicator of microbial metabolism and play an important role in biogeochemistry of terrestrial ecosystems^79^. We found that with the increase of vegetation degradation intensity and soil depth, soil catalase and amylase enzyme activity decreased significantly (Fig. 6b,c), which agreed with our second and third hypothesis about the dynamics of soil enzyme activities. This is likely a result of the decreases in aboveground biomass and soil nutrient sources that are a consequence of increased vegetation degradation level. Another reason could be the sharp decreases in SOC (Fig. 2) and underground biomass that occur with increasing soil depth and plant roots. Soil nutrient availability declines, and microbial activity and reproduction decrease with increasing soil depth^80^. In other words, the reduction in SOC content and plant roots with increasing soil depth would frequently cause decreased enzyme activity^81^.

Urease activity decreased with increasing soil depth (Fig. 6a). The urease activity was mainly concentrated in the 0–20 cm layer. Urease activity in this surface layer of CK and LD was significantly higher than that in the MD and HD wet meadows. This result may be due to the higher aboveground biomass of CK and LD wet meadows, the higher root growth in the 0–20 cm layer increasing soil permeability, and the higher SOC increasing the activity of soil microorganisms^82^.

Soil amylase activity reached the highest level in summer under all four degradation levels, and there was no significant difference in soil amylase activity in spring, autumn, and winter (*P* > 0.05, Fig. 8). This may be due to the abundant rainfall in summer, and the increase in soil temperature and moisture that increase the speed of soil carbon cycle and improve the ability of soil microorganisms to metabolize enzymes^83^. In addition, even though soil nutrient content is higher in spring, surface soil freezing during spring limits the oxygen content in the soil, and the aerobic microbial activity decreases^84^, resulting in lower amylase activity in spring than summer.

Catalase activity in the 0–100 cm soil profile was the highest in spring (Fig. 9) under all four degradation levels. Compared with summer, the activity of catalase in autumn increased slightly, but there was no significant difference (n = 16, P = 0.138). Catalase activity decreased to the minimum in winter. This may be because the soil began to thaw in spring. The nutrients and intracellular enzymes in the cells of soil organisms (soil animals, microorganisms, and roots) and plant debris that are lethal to freezing and thawing are released into the soil^85,86^, and the soil microorganisms start to be activated. However, because the surface freezing restricts the entry of oxygen into the soil, in order to reduce the toxic effect of soil hypoxia on aerobic microorganisms, the activity of soil catalase is increased only to a certain extent^87^. In winter, soil temperatures are low or the soil is frozen, resulting in decreased soil microbial activity and minimum soil catalase activity.

Soil urease activity showed a fluctuating decrease trend over time (Fig. 7). The variation of soil urease activity and TN content was consistent in spring and winter, but opposite in summer and autumn (Fig. 5a). Urease (urea amidohydrolase) supplies nitrogen to plants from natural and fertilizer sources. Plants grow most vigorouslyin summer. Thus plants require large amounts of nitrogen for their own cell synthesis^88^. In order to meet the absorption of soil available nitrogen, the activity of soil urease hydrolysis nitrogen increased. At the same time, soil urease is involved in urea hydrolysis, while most of the enzyme activity exists in extracellular enzymes^89,90^. The freezing and thawing cycle in spring resulted in the fragmentation of aggregates and the breakdown of microbial cells, thereby increasing the release of intracellular enzymes to the soil. These actions also increased the contact area between microorganisms and active organic matter, and the energy substances required for microbial metabolism. Furthermore, soil urease activity in the 0–10 cm layer increased in autumn, likely due to the lower soil temperature and active microbial metabolism in autumn caused by the heavy rainfall^91^.

### Interaction between soil nutrients and enzyme activities

Soil enzymes interact with the soil environment to indirectly affect soil nutrient levels. Soil enzymes are the main driver of soil decomposition processes. Previous studies have shown that increased soil temperature can increase soil enzyme activity^92–95^, but there are some differences in the temperature sensitivity for different ecosystems and different kinds of soil enzymes^96,97^. Soil carbon turnover and nutrient cycling depend on soil enzyme activity^98^. The results of our study showed that SOC and TN were positively correlated with soil catalase and amylase (*P* < 0.05), and negatively correlated with soil urease (*P* < 0.05). These empirical findings agree with many other studies that found significant correlations between soil enzyme activities and soil nutrients such as SOC and soil TN^99,100^. At the same time, soil enzymatic activities can indicate the ecosystem function of the soil because they can reflect the intensity and direction of various biochemical processes occurring in soil, and also correlate closely with soil fertility^101,102^. Given that soil enzymes directly participate in the circulation of material and energy in soil, and in the process of soil metabolism with soil microorganisms, they play an important role in promoting soil evolution and formation, material circulation, and energy flow^103,104^. Soil enzyme activity is affected by a series of biological factors such as microbial biomass and enzyme synthesis and secretion, and by abiotic factors such as pH and substrate availability^102^. Other factors affecting soil enzyme activity include vegetation type, root system, soil animals, litter quality, soil aggregates, climate, altitude, and human disturbance^105,106^.

## Conclusions

In this study, vertical and seasonal variations of soil nutrients and enzyme activities were compared among wet meadow systems with four levels of vegetation degradation in Qinghai-Tibet Plateau. We found that vegetation degradation levels significantly affected the vertical and seasonal distribution of soil nutrients (SOC, TN, and TP) and enzyme activities (urease, catalase, and amylase). Among the four vegetation degradation levels, the primary wet meadow (CK) had a significantly higher SOC, TN, catalase, and amylase activities throughout the year. In addition, the greatest differences in soil nutrients and enzyme activities were observed in surface soil, and considerable variations were also observed in deeper soil layers. The results show that vegetation degradation reduces soil carbon storage and nutrient cycling capacity. Overall, this study provides valuable information on the seasonal and vertical dynamics of soil nutrients and enzyme activities in Qinghai-Tibet Plateau and how they may be affected by vegetation degradation. Moreover, this year-long study suggests that the dynamics of soil nutrients and enzyme activities in wet meadows highly depends on time and depth and therefore require a longer timescale for further research to better understand their season dynamics. Further, our findings confirm the role that vegetation degradation plays in the dynamics of soil nutrients and enzyme activities at alpine wet meadows. Vegetation degradation will be seen more apparently at alpine wet meadows in the Qinghai-Tibet Plateau in the future under climate change and more intensified human activities. Therefore, more studies are needed for us to better understand the dynamics of soil nutrients and enzyme activities and how they would affect the health and stability of alpine wet meadows in the Qinghai-Tibet Plateau.

## Supplementary information

Supplementary information.
