# Supplementary material for: Vegetation degradation impacts soil nutrients and enzyme activities in wet meadow on the Qinghai-Tibet Plateau
Source: Sci Rep. 2020 Dec 4;10:21271. doi: 10.1038/s41598-020-78182-9 (PMC7718246; doi:10.1038/s41598-020-78182-9)
Supplement: Supplementary file 1 — Supplementary information. [file 41598_2020_78182_MOESM1_ESM.pdf]

Title: Vegetation degradation impacts soil nutrients and enzyme activities in wet meadow on the Qinghai-Tibet Plateau

Jiangqi Wu[1a], Haiyan Wang[1a], Guang Li[*a], Weiwei Ma[a], Jianghua Wu[*b], Yu Gong[b], Guorong Xu[a]

[a]College of Forestry, Gansu Agricultural University, Lanzhou, 730070, China.

[b]School of Science and the Environment, Memorial University of Newfoundland, 20 University Drive, Corner Brook, NL, A2H 5G4, Canada.

*Corresponding Author: Guang Li and Jianghua Wu

e-mail: 1462528657@qq.com; jwu@grenfell.mun.ca

Table S1. Results of a repeated-measures ANOVA testing for differences in soil organic carbon (SOC), total nitrogen (TN) and total phosphorous (TP) among vegetation types using season as the repeated variable. VD: vegetation degradation, S: season.

Source of variation		SOC			TN			TP		
		df	F	P	df	F	P	df	F	P
VD		3	639.660	.000	3	419.511	.000	3	74.381	.000
S	0-10cm	3	70.656	.000	3	274.508	.000	3	1235.414	.000
VD×S		9	19.854	.000	9	93.499	.000	9	93.248	.000
VD		3	287.513	.000	3	176.857	.000	3	271.974	.000
S	10-20cm	3	12.101	.000	3	94.363	.000	3	747.918	.000
VD×S		9	59.780	.000	9	35.859	.000	9	45.191	.000
VD		3	53.434	.000	3	147.948	.000	3	66.090	.000
S	20-40cm	3	7.013	.000	3	291.738	.000	3	517.868	.000
VD×S		9	29.913	.000	9	119.193	.000	9	26.532	.000
VD		3	2.585	.070	3	13.719	.000	3	164.981	.000
S	40-60cm	3	8.944	.000	3	1335.622	.000	3	754.678	.000
VD×S		9	22.772	.000	9	63.745	.000	9	55.453	.000
VD		3	10.240	.000	3	32.953	.000	3	44.600	.000
S	60-80cm	3	59.929	.000	3	1859.819	.000	3	410.650	.000
VD×S		9	16.826	.000	9	69.662	.000	9	68.981	.000
VD		3	21.679	.000	3	19.286	.000	3	58.463	.000
S	80-100cm	3	10.310	.000	3	866.909	.000	3	1113.460	.000
VD×S		9	8.194	.000	9	19.031	.000	9	155.605	.000

Table S2. Results of a repeated-measures ANOVA testing for differences in soil urease, catalase and amylase activity among vegetation types using season as the repeated variable. VD: vegetation degradation, S: season.

Source of variation		urease			catalase			amylase		
		df	F	P	df	F	P	df	F	P
VD		3	321.865	.000	3	488.904	.000	3	744.023	.000
S	0-10cm	3	84.330	.000	3	67.498	.000	3	386.428	.000
VD×S		9	14.061	.000	9	16.892	.000	9	129.886	.000
VD		3	562.991	.000	3	287.372	.000	3	126.914	.000
S	10-20cm	3	17.557	.000	3	20.242	.000	3	4.171	.013
VD×S		9	29.873	.000	9	4.569	.000	9	12.469	.000
VD		3	505.920	.000	3	309.800	.000	3	341.064	.000
S	20-40cm	3	.717	.549	3	29.971	.000	3	8.474	.000
VD×S		9	51.956	.000	9	5.288	.000	9	10.292	.000

VD		3	378.888	.000	3	352.193	.000	3	475.764	.000
S	40-60cm	3	100.935	.000	3	46.872	.000	3	6.046	.002
VD×S		9	25.069	.000	9	6.132	.000	9	11.548	.000
VD		3	89.566	.000	3	164.334	.000	3	245.937	.000
S	60-80cm	3	195.676	.000	3	25.789	.000	3	9.937	.000
VD×S		9	24.389	.000	9	10.185	.000	9	9.339	.000
VD		3	169.352	.000	3	137.697	.000	3	237.440	.000
S	80-100cm	3	282.459	.000	3	18.184	.000	3	26.733	.000
VD×S		9	21.804	.000	9	11.299	.000	9	24.638	.000
